# Joint Power Control and Resource Allocation for Optimizing the D2D User Performance of Full-Duplex D2D Underlying Cellular Networks

**DOI:** 10.3390/s23239549

**Published:** 2023-12-01

**Authors:** Yuetian Zhou, Bowen Cai, Xue Ding

**Affiliations:** 1 Department of Mobile Communication and Terminal Technology, China Telecom Research Institute, Beijing 100032, Chinadingx@chinatelecom.cn (X.D.); 2 National Key Laboratory of Science and Technology on Communications, University of Electronic Science and Technology of China, Chengdu 610056, China

**Keywords:** full-duplex, underlaid D2D, Kuhn–Munkres algorithm, uniform algorithm, nonlinear programming

## Abstract

D2D communication is a promising technology for enhancing spectral efficiency (SE) in cellular networks, and full-duplex (FD) has the potential to double SE. Due to D2D’s short-distance communication and low transmittance power, it is natural to integrate FD into D2D, creating FD-D2D to underlay a cellular network to further improve SE. However, the residual self-interference (RSI) resulting from FD-D2D and interference arising from spectrum sharing between D2D users (DUs) and cellular users (CUs) can restrict D2D link performance. Therefore, we propose an FD-D2D underlying cellular system in which DUs jointly share uplink and downlink spectral resources with CUs. Moreover, we present two algorithms to enhance the performance experience of DUs while improving the system’s SE. For the first algorithm, we tackle an optimization problem aimed at maximizing the sum rate of FD-DUs in the system while adhering to transmittance power constraints. This problem is formulated as a mixed-integer nonlinear programming problem (MINLP), known for its mathematical complexity and NP-hard nature. In order to address this MINLP, our first algorithm decomposes it into two subproblems: power control and spectral resource allocation. The power control aspect is treated as a nonlinear problem, which we solve through one-dimensional searching. Meanwhile, spectral resource allocation is achieved using the Kuhn–Munkres algorithm, determining the pairing of CUs and DUs sharing the same spectrum. As for the second algorithm, our objective is to enhance the individual performance of FD-DUs by maximizing the minimum rate among them, ensuring more uniform rate performance across all FD-DUs. In order to solve this optimization problem, we propose a novel spectral resource allocation algorithm based on bisection searching and the Kuhn–Munkres algorithm, whereas the power control aspect remains the same as in the first algorithm. The numerical results demonstrate that our proposed algorithm effectively enhances the performance of DUs in an FD-D2D underlying cellular network when compared to the sum rate maximization design.

## 1. Introduction

With the tremendous growth in mobile devices and traffic, the spectrum has become more and more scarce. Since the D2D communication underlying the cellular networks can share the same spectrum with cellular users and offload traffic to alleviate base station pressure, it has been regarded as a promising technology to address the issue of spectrum scarcity [1]. However, reusing the spectrum alongside cellular users will introduce mutual interference, which sometimes results in the degradation of system capacity rather than improving it. In order to tackle this challenging problem, D2D underlying networks have attracted significant research interest to interference management, resource allocation, and power control to enhance network performance [2,3,4,5,6,7,8]. Reference [3] proposes a hypergraph-based channel allocation method to maximize the cell’s sum rate, allowing multiple D2D pairs to share uplink channels with cellular users. Reference [4] introduces a downlink subcarrier reuse technique based on convex optimization. In addition, in reference [5], DUs collaboratively reuse uplink and downlink spectrum resources with CUs through a two-step scheme involving power allocation and user pairing, resulting in a higher system capacity than with uplink and downlink sharing alone. Reference [6] develops centralized and distributed power control algorithms for a random network model of underlying D2D. In reference [7], NOMA technology was employed to overcome the mutual interference caused by spectrum sharing in D2D underlying networks. Moreover, the aforementioned papers primarily focus on one-to-one scenarios where a DU can only reuse subcarrier resources from one CU and vice versa. However, reference [8] introduces a joint uplink sharing and power allocation scheme to maximize the sum rate of DUs in a many-to-many scenario. In this scenario, a subcarrier can be shared by multiple DUs, and a DU can reuse multiple subcarriers. Nevertheless, as the network permits multiple D2D pairs to reuse the same subcarrier in this scenario, mutual interference intensifies, potentially leading to capacity degradation [9,10]. Therefore, investigating more efficient resource co-ordination schemes to enhance system performance becomes a nontrivial task.

In recent times, full-duplex (FD) technology has garnered significant attention, particularly with advancements in self-interference (SI) cancellation techniques, and it is now being considered as a candidate technology for 6G networks. A three-level architecture has been proposed to compartmentalize SI cancellation into three distinct domains: the antenna domain, analog domain, and digital domain. When these three levels of architecture are collectively applied to mitigate SI, the SI cancellation capability can reach up to 125 dB [11,12,13]. Despite remarkable progress in SI cancellation technologies, achieving complete SI elimination remains a challenging endeavor. In the study conducted in reference [14], the integration of D2D communication with FD base stations was investigated to enhance spectral efficiency (SE). The numerical results clearly demonstrate that performance improvements are significantly affected by the presence of strong residual self-interference (RSI). It is worth noting that in addition to SI cancellation capability, the magnitude of RSI is closely linked to the transmit power of FD devices, with lower transmit power levels resulting in reduced RSI. Given that D2D communication is inherently a short-distance technology, where devices operate at low transmit power levels, it is only natural to integrate FD into D2D communications.

When compared to traditional half-duplex (HD) D2D communications, FD-D2D offers the potential to significantly enhance system spectral efficiency (SE) and reduce latency. This improvement is due to the simultaneous transmission of uplink and downlink signals. As a result, recent research efforts have been dedicated to exploring FD-D2D communication. In the realm of FD-D2D research, several distinct scenarios have been explored. References [15,16] delve into the fundamental scenario involving a single FD-D2D pair and a single CU. Specifically, ref. [15] provides a closed-form approximation for the sum rate. Additionally, the focus shifts to multi-user scenarios in references [17,18,19]. In [17], both perfect and statistical CSI estimations are considered, leading to the proposal of a heuristic algorithm that maximizes the sum rate for cellular uplink sharing. This algorithm employs 2D global searching and the Kuhn–Munkres algorithm. The numerical findings in ref. [18] reveal that, with sufficient SI cancellation, FD-D2D underlying systems offer significantly greater capacity gains compared to traditional HD-D2D systems. Meanwhile, ref. [19] presents centralized and distributed power control mechanisms designed to maximize D2D link throughput. These mechanisms take into account the locations of homogeneous spatial Poisson point processes for FD-D2D users. Moreover, there are other promising techniques being considered that coexist with FD-D2D, such as FD-D2D underlying cellular networks equipped with base station MIMO antennas, as explored in [20,21]. However, the previous studies typically concentrate on overall cell performance improvement, with only a limited number of works focusing on maximizing D2D link throughput, as discussed in [19]. Due to the introduced mutual interference and RSI, achieving the expected capacity gains for FD-DUs becomes challenging. Therefore, to offload more traffic and alleviate pressure on CUs, there is an urgent need for interference management algorithms to enhance the performance of FD-DUs.

In this paper, we present two efficient algorithms aimed at enhancing the user performance experience for DUs within FD-D2D underlying cellular networks with multiple users, including both CUs and DUs. The primary contributions of this study are outlined as follows:

1. For the first algorithm, our objective is to maximize the sum rate of DUs in an FD-D2D underlying system to enhance the performance of FD-DUs. This optimization problem is formulated as a MINLP problem, which is then decomposed into two subproblems: power control and resource allocation. In the first subproblem, we determine the optimal power allocation that maximizes the rate of each DU in the spectrum sharing of each CU-DU pair. This is achieved through a one-dimensional search within a finite set of power levels. Next, in the second subproblem of resource allocation, we employ the Kuhn–Munkres maximal weight method to identify the optimal pairing of each DU with a CU for the purpose of maximizing the sum rate of all DUs within the cell. The first algorithm is referred to as MaxSumDU-OP (maximizing sum rate of dus with optimal power and optimal pairing) in the following sections of this paper.
2. The MaxSumDU-OP algorithm focuses on maximizing the overall sum rate for DUs, but it does not guarantee uniform user performance for each individual D2D pair. In order to address this limitation, we introduce the second algorithm, which aims to maximize the minimum rate among all D2D pairs. This approach ensures a more uniform user performance experience across all DUs. What sets the second algorithm apart from MaxSumDU-OP is its approach to solving the resource allocation subproblem. We have developed a uniform performance algorithm for this purpose, which utilizes bisection searching and multiple iterations of the Kuhn–Munkres method. This approach helps identify optimal sharing pairings to guarantee individual user experiences for D2D pairs. We denote this second algorithm as MaxMinDU-OP (maximizing the minimum rate of D2D user pairs with optimal power and optimal pairing).
3. The two proposed algorithms jointly share uplink and downlink spectrum resources, aiming to achieve higher system capacity gains while ensuring the performance of D2D links.

The remainder of the paper is organized as follows: Section 2 introduces the system model of FD-D2D underlying cellular networks. Section 3 describes the MaxSumDU-OP algorithm for maximizing the sum rates of FD-DUs, and Section 4 presents the MaxMinDU-OP algorithm to provide a uniform experience for DUs by maximizing the minimum rate among DUs. In Section 5, we provide numerical results to validate the efficiency of the proposed algorithm. Finally, Section 6 concludes the paper.

## 2. System Model

**Figure 1:**
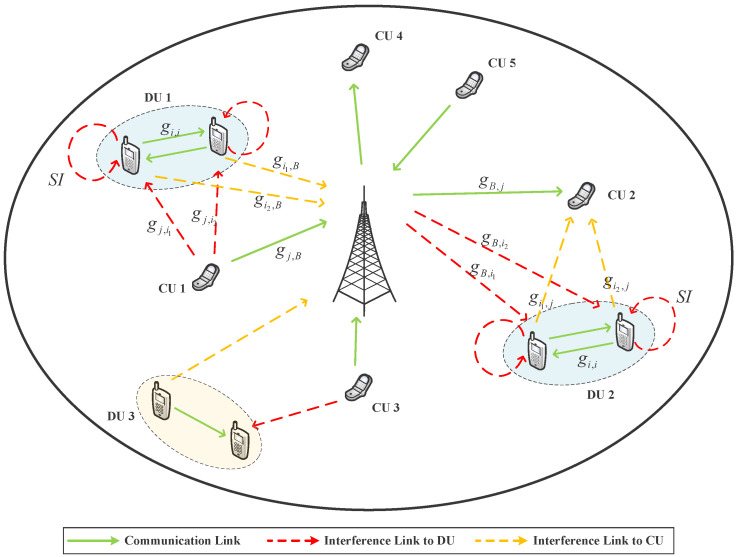
The cellular system incorporates various kinds of D2D within the cell.

The diagram in Figure 1 illustrates a cellular environment with various D2D underlying networks. In this representation, the base station (BS) is positioned at the center, with multiple DUs and CUs randomly distributed throughout the cell. Three categories of spectrum usage exist within this cell. The first category comprises DUs operating in FD mode, reusing subcarriers in pairs like DU1-CU1 and DU2-CU2. DU1-CU1 reuses uplink spectrum resources, whereas DU2-CU2 shares downlink spectrum resources. The second category represents a traditional scenario, exemplified by the DU3-CU3 pair, where both DUs and CUs operate in half-duplex (HD) mode, sharing resources. The final category includes unpaired CUs, denoted as CU4 and CU5, which independently utilize allocated resources. Since FD-D2D can nearly double the SE of DUs, this paper focuses solely on systems composed of FD-DUs and CUs. CUs are assumed to operate using the traditional half-duplex frequency-division duplex (FDD) mode, with the ability for FD-D2D pairs to jointly reuse uplink and downlink spectrum resources with them.

We denote the distributed DUs and CUs in the cell as DU*i* and CU*j*, respectively, where $i\in D=\left\{1,2,\ldots ,S\right\}$ and $j\in C=\left\{1,2,\ldots ,T\right\}$. The two distinct devices in a D2D pair are labeled as $i_{1}$ and $i_{2}$. Additionally, we assume that CUs utilize the entire carrier resource, which is evenly pre-allocated. In order to simplify the complexity arising from intricate interference scenarios, we exclusively consider the “one-to-one” scenario. In this scenario, each subcarrier can be shared by, at most, one DU, and each DU can reuse, at most, one subcarrier. These straightforward assumptions establish a robust foundation for addressing more challenging issues within the FD-D2D system in complex scenarios.

The channels are modeled to incorporate path loss, slow shadowing, and fast fading. For example, the channel gain between CU*j* and BS, denoted as $g_{j,B}$, is formulated as

(1) $$g_{j,B}=G\beta_{j,B}\Gamma_{j,B}l_{j,B}^{-\alpha }$$

where the primary notations and definitions are listed in Table 1.

Accordingly, the gain of other channels described in Figure 1 is notated as $g_{i_{1},B}$, $g_{i_{2},B}$, $g_{B,i_{1}}$, $g_{B,i_{2}}$, $g_{i_{1},j}$, $g_{i_{2},j}$, $g_{j,i_{1}}$, $g_{j,i_{2}}$, $g_{B,j}$, and $g_{i,i}$, respectively. In particular, bidirectional links within FD-D2D pairs operate simultaneously on the same frequency. Therefore, the gain for both directions is denoted as $g_{i,i}$.

In order to establish a pairing between DU*i* and CU*j*, we introduce a binary variable, denoted as $\rho_{i,j}$, to serve as a pairing indicator. If DU*i* shares the same subcarrier with CU*j*, then we set $\rho_{i,j}$ to 1; otherwise, it is set to 0. Specifically, we use $\rho_{i,j}^{u}$ for the uplink scenario and $\rho_{i,j}^{d}$ for the downlink scenario. Figure 1 illustrates the interference scenario of FD-D2D, which is notably more complex compared to the traditional HD setup. This complexity primarily arises from the presence of RSI caused by FD communication. In order to address this intricacy, we divide the problem into two distinct cases, each involving the reuse of either the uplink or downlink resources of the CUs. In the context of reusing uplink resources, the signal-to-interference plus noise ratio (SINR) experienced by CU*j* and DU*i* can be mathematically expressed as follows:

(2) $$\gamma_{j}^{u}=\frac{p_{j}^{}g_{j,B}^{}}{\sum_{i=1}^{S}\rho_{i,j}^{u}p_{i}\left(g_{i_{1},B}+g_{i_{2},B}\right)+N_{0}}$$

(3a) $$\begin{array}{cc} \gamma_{i_{1}}^{u} & =\frac{p_{i}g_{i,i}}{\sum_{j=1}^{T}\rho_{i,j}^{u}\left(p_{j}g_{j,i_{1}}+p_{i}\cdot \eta \right)+N_{0}} \end{array}$$

(3b) $$\begin{array}{cc} \gamma_{i_{2}}^{u} & =\frac{p_{i}g_{i,i}}{\sum_{j=1}^{T}\rho_{i,j}^{u}\left(p_{j}g_{j,i_{2}}+p_{i}\cdot \eta \right)+N_{0}} \end{array}$$

where $p_{i}$ and $p_{j}$ represent the transmit power of DU*i* and CU*j*, respectively. The parameter η represents self-interference cancellation (SIC) capability, and $N_{0}$ denotes the variance of zero-mean additive white gaussian noise (AWGN). When examining the scenario of downlink resource reuse, the SINR for CU*j* and DU*i* can be expressed as follows:

(4) $$\gamma_{j}^{d}=\frac{p_{B,j}g_{B,j}^{}}{\sum_{i=1}^{S}\rho_{i,j}^{d}p_{i}\left(g_{i_{1},j}+g_{i_{2},j}\right)+N_{0}}$$

(5a) $$\begin{array}{cc} \gamma_{i_{1}}^{d} & =\frac{p_{i}g_{i,i}}{\sum_{j=1}^{T}\rho_{i,j}^{d}\left(p_{B,j}g_{B,i_{1}}+p_{i}\cdot \eta \right)+N_{0}} \end{array}$$

(5b) $$\begin{array}{cc} \gamma_{i_{2}}^{d} & =\frac{p_{i}g_{i,i}}{\sum_{j=1}^{T}\rho_{i,j}^{d}\left(p_{B,j}g_{B,i_{2}}+p_{i}\cdot \eta \right)+N_{0}} \end{array}$$

where $P_{B,j}$ signifies the transmit power from the BS to CU*j*.

Hence, the achievable rates for both the uplink and downlink of CU*j* and its corresponding DU*i* can be determined separately as follows:

(6) $$R_{j}^{u}=\log_{2}\left(1+\gamma_{j}^{u}\right)$$

(7) $$R_{j}^{d}=\log_{2}\left(1+\gamma_{j}^{d}\right)$$

(8a) $$\begin{array}{cc} \; & R_{i_{1}}^{u}=\log_{2}\left(1+\gamma_{i_{1}}^{u}\right) \end{array}$$

(8b) $$\begin{array}{cc} \; & R_{i_{2}}^{u}=\log_{2}\left(1+\gamma_{i_{2}}^{u}\right) \end{array}$$

(9a) $$\begin{array}{cc} \; & R_{i_{1}}^{d}=\log_{2}\left(1+\gamma_{i_{1}}^{d}\right) \end{array}$$

(9b) $$\begin{array}{cc} \; & R_{i_{2}}^{d}=\log_{2}\left(1+\gamma_{i_{2}}^{d}\right) \end{array}$$

## 3. FD-DUs Capacity Maximization Design

In this section, to ensure the optimal performance of DUs in the one-to-one scenario of the FD-D2D system, we propose a two-step heuristic algorithm. This algorithm involves power control and resource allocation to maximize the total rate of *S* DUs within the cell while ensuring that each link meets the minimum achievable rate requirement.

### 3.1. Problem Formulation

The CU in the cell operates in either the uplink or downlink mode. For the sake of convenience in notation, we denote CU*j*’s rate as

(10) $$R_{j}=\beta_{j}R_{j}^{u}+\left(1-\beta_{j}\right)R_{j}^{d}$$

where $\beta_{j}\in B=\left\{\beta_{j}|\beta_{j}=0\;or\;1,j=1,2,\ldots ,T\right\}$ represents an indicator of uplink or downlink operation. Specifically, if the $\beta_{j}=1$, when $\beta_{j}=1$, CU*j* functions as an uplink user; otherwise, it operates as a downlink user.

In order to obtain the maximum sum rate of FD-DUs, the optimization problem is formulated as follows:

(11) $$\begin{array}{cc} P1:\;\;\;\; & \max_{\begin{array}{c} \rho_{i,j},\;p_{i}\\ p_{j},\;p_{B,j} \end{array}}\;\sum_{i=1}^{S}R_{i_{1}}+R_{i_{2}} \end{array}$$

(11a) $$\begin{array}{cc} s.t.\;\;\;\;\; & \gamma_{j}^{u}\geq \gamma_{j}^{u,\mathrm{req}},\;\gamma_{j}^{d}\geq \gamma_{j}^{d,\mathrm{req}},\;\;\forall j\in C \end{array}$$

(11b) $$\begin{array}{cc} \; & \gamma_{i_{1}}^{u}\geq \gamma_{i}^{\mathrm{req}},\;\gamma_{i_{2}}^{u}\geq \gamma_{i}^{\mathrm{req}}\;\;\forall i\in D \end{array}$$

(11c) $$\begin{array}{cc} \; & \gamma_{i_{1}}^{d}\geq \gamma_{i}^{\mathrm{req}},\;\gamma_{i_{2}}^{d}\geq \gamma_{i}^{\mathrm{req}}\;\;\forall i\in D \end{array}$$

(11d) $$\begin{array}{cc} \; & 0\leq p_{i}\leq p_{i}^{\max},\;\;\forall i\in D \end{array}$$

(11e) $$\begin{array}{cc} \; & 0\leq p_{j}\leq p_{j}^{\max},\;\;\forall j\in C \end{array}$$

(11f) $$\begin{array}{cc} \; & 0\leq p_{B,j}\leq p_{B,j}^{\max},\;\;\forall j\in C \end{array}$$

(11g) $$\begin{array}{cc} \; & \left(\sum_{i=1}^{S}\rho_{i,j}^{d}\right)\left(\sum_{i=1}^{S}\rho_{i,j}^{u}\right)=0,\;\;\forall j\in C \end{array}$$

(11h) $$\begin{array}{cc} \; & \left(\sum_{j=1}^{T}\rho_{i,j}^{d}\right)\left(\sum_{j=1}^{T}\rho_{i,j}^{u}\right)=0,\;\;\forall i\in D \end{array}$$

(11i) $$\begin{array}{cc} \; & \rho_{i,j}^{d},\rho_{i,j}^{u}\in \left\{0,1\right\},\;\;\forall i\in D,\;\forall j\in C \end{array}$$

for which constraints (11a)–(11c) ensure that the data rates for both the CU and DU links meet the specified access rate requirements, thereby satisfying quality of service (QoS). In this context, $\gamma_{j}^{u,\mathrm{req}}$, $\gamma_{j}^{d,\mathrm{req}}$, and $\gamma_{i}^{\mathrm{req}}$ represent the minimum required SINRs for the uplink and downlink links of CU*j* and DU*i*, respectively. Constraints (11d)–(11f) impose power limitations, where $p_{i}^{\max}$, $p_{j}^{\max}$, and $p_{B,j}^{\max}$ denote the maximum allowable transmit powers for DU*i*, CU*j*, and the BS, respectively. In order to ensure a “one-to-one” subcarrier sharing scenario, conditions (11g) and (11h) are employed. Specifically, (11g) guarantees that each pre-allocated subcarrier of CU*j* can be shared by, at most, one DU, whereas (11h) ensures that any DU*j* can reuse, at most, one subcarrier pre-allocated to the CUs. Certainly, P1 is a MINLP problem, which is mathematically intractable. Consequently, it is divided into separate subproblems for power control and resource allocation, which can be solved independently.

### 3.2. Power Control

In the power control phase, our goal is to determine the optimal transmit power for each DU-CU pair in order to maximize the data rate of the DU pair in DU-CU. In order to simplify the original problem P1, we introduce an optimization problem referred to as P2. This problem is specifically focused on a single CU*j*-DU*i* pair and is defined as follows:

(12) $$\begin{array}{cc} P2:\;\;\;\; & \max_{\begin{array}{c} \rho_{i,j},\;p_{i}\\ p_{j},\;p_{B,j} \end{array}}\;R_{i_{1},j}^{D}+R_{i_{2},j}^{D} \end{array}$$

(12a) $$\begin{array}{cc} s.t.\;\;\;\;\; & \gamma_{j}^{u}\geq \gamma_{j}^{u,\mathrm{req}},\;\gamma_{j}^{d}\geq \gamma_{j}^{d,\mathrm{req}} \end{array}$$

(12b) $$\begin{array}{cc} \; & \begin{array}{cc} \; & \gamma_{i_{1}}^{u}\geq \gamma_{i}^{\mathrm{req}},\;\gamma_{i_{2}}^{u}\geq \gamma_{i}^{\mathrm{req}},\\ \; & \gamma_{i_{1}}^{d}\geq \gamma_{i}^{\mathrm{req}},\;\gamma_{i_{2}}^{d}\geq \gamma_{i}^{\mathrm{req}} \end{array} \end{array}$$

(12c) $$\begin{array}{cc} \; & 0\leq p_{i}\leq p_{i}^{\max} \end{array}$$

(12d) $$\begin{array}{cc} \; & 0\leq p_{j}\leq p_{j}^{\max} \end{array}$$

(12e) $$\begin{array}{cc} \; & 0\leq p_{B,j}\leq p_{B,j}^{\max} \end{array}$$

where $R_{i_{1},j}^{D}$ and $R_{i_{2},j}^{D}$ denote the rate of the CU within the CU*j*-DU*i* pair. It is evident that P2 represents a nonlinear programming problem that can be solved using geometric programming techniques. Since D2D communications typically involve short-distance links, we adopt the strategy of setting $g_{j,i_{1}}=g_{j,i_{2}}=g_{j,i}$, where $g_{j,i}$ represents the channel gain between CU*j* and the midpoint of the two devices in the D2D connection. In order to manage computational complexity, we address P2 separately for the uplink and downlink sharing scenarios. As an example, for uplink sharing, we can derive $\gamma_{i_{1}}^{u}=\gamma_{i_{2}}^{u}=\gamma_{i}^{u}$ from (3a) and (3b), where $\gamma_{i}^{u}$ represents the SINR for D2D when the uplink subcarrier of CU is reused, thereby obtaining $R_{i_{1}}^{u}=R_{i_{2}}^{u}=R_{i}^{u}$, that is, the objective function in P2 can transfer to $2R_{i,j}^{D}$.

**Figure 2:**
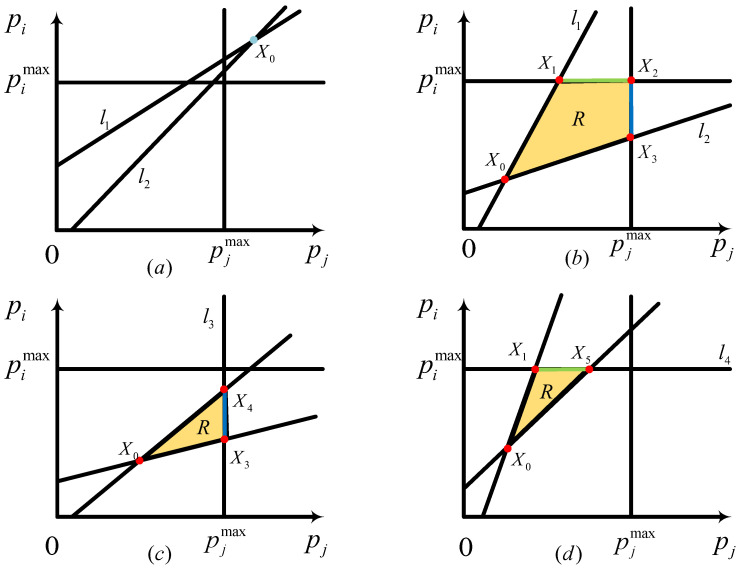
Feasible region for power control algorithm of the pair DU*i*-CU*j*.

Based on the constraints (12a)–(12e), in Figure 2, we have illustrated the following regions enclosed by $l_{1}$ corresponds to $\gamma_{j}^{u}=\gamma_{j}^{u,\mathrm{req}}$, $l_{2}$ pertains to $\gamma_{i}^{u}=\gamma_{i}^{\mathrm{req}}$, $l_{3}$ indicates $p_{j}=p_{j}^{\max}$, and $l_{4}$ represents $p_{i}=p_{i}^{\max}$. The defined region, R, encompasses the feasible domain for the power allocation of CU*j* and DU*i*. Our objective is to find the optimal power solution ($p_{i}$, $p_{j}$) within the region R to maximize $2R_{i,j}^{D,u}$. It is important to note that R can either be an empty set, as shown in Figure 2a, or a closed set, as depicted in Figure 2b–d. In the case of a non-empty set, the optimal power solution ($p_{i}^{opt}$, $p_{j}^{opt}$) must lie within R and is assumed to be below the maximum allowable power. We substitute ($\lambda p_{i}^{opt}$, $\lambda p_{j}^{opt}$) for ($p_{i}^{opt}$, $p_{j}^{opt}$) in the objective function of P2. For any λ>1 and $\lambda \in R^{+}$, we deduce to obtain the following:

(13) $$\begin{array}{cc} 2R_{i,j}^{D,u}\left(\lambda p_{i}^{opt},\lambda p_{j}^{opt}\right)\; & =log_{2}{\left(1+\frac{p_{i}^{opt}g_{i,i}}{p_{j}^{opt}g_{j,i}+p_{i}^{opt}\cdot \eta +\left(N_{0}/\lambda \right)}\right)}^{2}\\ \; & >log_{2}{\left(1+\frac{p_{i}^{opt}g_{i,i}}{p_{j}^{opt}g_{j,i}+p_{i}^{opt}\cdot \eta +N_{0}}\right)}^{2}\\ \; & =2R_{i,j}^{D,u}\left(p_{i}^{opt},p_{j}^{opt}\right) \end{array}$$

Since $R_{j}^{D,u}\left(\lambda p_{i}^{op},\lambda p_{j}^{op}\right)>R_{j}^{D,u}\left(p_{i}^{op},p_{j}^{op}\right)$ for any λ>1, it is evident that this contradicts the assumption that ($p_{i}^{opt}$, $p_{j}^{opt}$) is the optimal power solution. Hence, at least one component of the optimal power solution $\left(p_{i}^{opt},p_{j}^{opt}\right)$ must reach the maximum value $p_{i}^{\max}$ or $p_{j}^{\max}$. Consequently, Theorem 1 is derived.

**Theorem** **1.** *At least one component of the optimal power solution equals the maximum value, specifically, the optimal solution is ($p_{i}^{opt}$, $p_{j}^{opt}$) where either $p_{i}^{opt}=p_{i}^{\max}$ or $p_{j}^{opt}=p_{j}^{\max}$.*

Theorem 1 establishes that the optimal solution exists at the boundaries of the feasible region. As depicted in Figure 2, there are four potential scenarios for the feasible region R, each determined by different maximal transmit power, channel gain, and SINR requirements. The optimal solution can be found along the line segments $\bar{X_{1}X_{2}}$, $\bar{X_{2}X_{3}}$, $\bar{X_{3}X_{4}}$, or $\bar{X_{1}X_{5}}$. In orde to further identify the set of possible optimal power solutions, we need Theorem 2, as presented below. Theorem 2 has been established and documented in [2,5].

**Theorem** **2.** *If the feasible region R is bounded, the optimal power solution $\left(p_{i}^{opt},p_{j}^{opt}\right)$ may exclusively be located at the corners of R.*

According to the previously discussed theorem, the potential objective points of the optimal power solution can be marked in Figure 2, designated as $X_{1}$ to $X_{5}$. As detailed in ref. [2], the existence of solutions depends on the co-ordinates of $X_{0}$ and the slopes of $l_{1}$ and $l_{2}$. We label these points as $X_{0}$, $X_{1}$, $X_{2}$, $X_{3}$, $X_{4}$, and $X_{5}$, with co-ordinates $\left(p_{j}^{X_{0}},p_{i}^{X_{0}}\right)$, $\left(p_{j}^{X_{1}},p_{i}^{\max}\right)$, $\left(p_{j}^{\max},p_{i}^{\max}\right)$, $\left(p_{j}^{\max},p_{i}^{X_{3}}\right)$, $\left(p_{j}^{\max},p_{i}^{X_{4}}\right)$, and $\left(p_{j}^{X_{5}},p_{i}^{\max}\right)$, respectively. These values can be determined since $X_{1}$ to $X_{5}$ represent the intersections of lines $l_{1}$, $l_{2}$, $l_{3}$, and $l_{4}$. Therefore, we can identify the values of $p_{j}^{X_{0}}$, $p_{i}^{X_{0}}$, $p_{j}^{X_{1}}$, $p_{i}^{X_{3}}$, $p_{i}^{X_{4}}$, and $p_{j}^{X_{5}}$ as follows:

(14) $$P_{j}^{X_{0}}=\frac{\gamma_{j}^{u,\mathrm{req}}N_{0}\left(g_{i,i}-\gamma_{i}^{\mathrm{req}}\eta \right)+\gamma_{j}^{u,\mathrm{req}}\gamma_{i}^{\mathrm{req}}N_{0}\left(g_{i_{1},B}+g_{i_{2},B}\right)}{g_{j,B}\left(g_{i,i}-\gamma_{i}^{\mathrm{req}}\eta \right)-\gamma_{j}^{u,\mathrm{req}}\gamma_{i}^{\mathrm{req}}g_{i,i}\left(g_{i_{1},B}+g_{i_{2},B}\right)}$$

(15) $$\begin{array}{cc} P_{i}^{X_{0}}=\; & \frac{\gamma_{j}^{u,\mathrm{req}}N_{0}\left(g_{i,i}-\gamma_{i}^{\mathrm{req}}\eta \right)+\gamma_{j}^{u,\mathrm{req}}\gamma_{i}^{\mathrm{req}}N_{0}\left(g_{i_{1},B}+g_{i_{2},B}\right)}{g_{j,B}\left(g_{i,i}-\gamma_{i}^{\mathrm{req}}\eta \right)-\gamma_{j}^{u,\mathrm{req}}\gamma_{i}^{\mathrm{req}}g_{i,i}\left(g_{i_{1},B}+g_{i_{2},B}\right)}\\ \; & \times \frac{\gamma_{i}^{\mathrm{req}}g_{i,i}}{g_{i,i}-\gamma_{i}^{\mathrm{req}}\eta }+\frac{\gamma_{i}N_{0}}{g_{i,i}-\gamma_{i}^{\mathrm{req}}\eta } \end{array}$$

(16) $$\begin{array}{cc} P_{j}^{X_{1}}=\; & \frac{\gamma_{j}^{u,\mathrm{req}}\left[P_{i}^{\max}\left(g_{i_{1},B}+g_{i_{2},B}\right)+N_{0}\right]}{g_{j,B}} \end{array}$$

(17) $$\begin{array}{cc} P_{i}^{X_{3}}=\; & \frac{P_{j}^{\max}g_{j,B}-\gamma_{j}^{u,\mathrm{req}}N_{0}}{g_{i,i}-\gamma_{i}^{\mathrm{req}}\eta } \end{array}$$

(18) $$\begin{array}{cc} P_{i}^{X_{4}}=\; & \frac{P_{j}^{\max}g_{j,B}-\gamma_{j}^{u,\mathrm{req}}N_{0}}{\gamma_{j}^{u,\mathrm{req}}g_{i,B}} \end{array}$$

(19) $$\begin{array}{cc} P_{j}^{X_{5}}=\; & \frac{P_{i}^{\max}\left(g_{i,i}-\gamma_{i}^{\mathrm{req}}\eta \right)-\gamma_{i}^{\mathrm{req}}N_{0}}{\gamma_{j}^{u,\mathrm{req}}g_{i,i}} \end{array}$$

At this stage, we have obtained a finite set $X_{1},X_{2},X_{3},X_{4},X_{5}$ that comprises potential optimal solutions. This allows us to systematically search and compare all elements within the set to identify the maximum value of $Rj^{u}$. Consequently, the power control for the DU*i*-CU*j* pair in the uplink sharing scenario is resolved. Accordingly, in the case of the downlink, we can employ the same approach to resolve the power control and maximize $R{i,j}^{D,d}$.

### 3.3. Resource Allocation

We have presented the optimal power control method for each DU-CU pair, resulting in the maximal rate $R_{i,j}^{D}$. However, not every DU is paired with a corresponding CU. The unpaired DU cannot access the network, the rate of  which is 0. For unpaired CUs, we assume they operate at maximal transmit power; by taking uplink sharing as an example, the achieved rate for an unpaired CU*j* is denoted as

(20) $$R_{j}^{u,\mathrm{unp}}=\log_{2}\left(1+\frac{p_{j}^{\max}g_{j,B}}{N_{0}}\right)$$

When CU*j* pairs with DU*i*, the achieved rate of CU*j* will decrease due to the introduced mutual interference while DU*i* accesses the network and obtains the rate. In order to indicate this change, we denote the rate variation of them for uplink sharing as

(21) $$\begin{array}{cc} \; & \Delta R_{i,j}^{C,u}=R_{i,j}^{C,u}-R_{j}^{u,\mathrm{unp}} \end{array}$$

(22) $$\begin{array}{cc} \; & \Delta R_{i,j}^{D,u}=R_{i,j}^{D,u}-0 \end{array}$$

Similarly, the rate variation of CU*j* for the downlink can be represented as $\Delta R_{i,j}^{C,d}=R_{i,j}^{C,d}-R_{j}^{d,\mathrm{unp}}$ and $\Delta R_{i,j}^{D,d}=R_{i,j}^{D,d}$.

The optimal resource allocation scheme determines the pairing of CUs and DUs to achieve maximum capacity. This essentially poses an optimal user pairing problem, which can be cast as maximum-weight bipartite matching. Consequently, we can formulate P3 as follows:

(23) $$\begin{array}{cc} P3:\;\;\;\; & \max_{\rho_{i,j}}\sum_{j=1}^{T}\sum_{i=1}^{S}\;\left(\beta_{j}\rho_{i,j}^{u}\Delta R_{i,j}^{D,u}+\left(1-\beta_{j}\right)\rho_{i,j}^{d}\Delta R_{i,j}^{D,d}\right) \end{array}$$

(23a) $$\begin{array}{cc} s.t.\;\;\;\;\; & \left(\sum_{j=1}^{T}\rho_{i,j}^{d}\right)\left(\sum_{j=1}^{T}\rho_{i,j}^{u}\right)=0,\;\;\forall i\in D \end{array}$$

(23b) $$\begin{array}{cc} \; & \left(\sum_{i=1}^{S}\rho_{i,j}^{d}\right)\left(\sum_{i=1}^{S}\rho_{i,j}^{u}\right)=0,\;\;\forall j\in C \end{array}$$

(23c) $$\begin{array}{cc} \; & \rho_{i,j}^{u},\rho_{i,j}^{d}\in \left\{0,1\right\},\;\;\forall i\in D,\;\;\forall j\in C \end{array}$$

(23d) $$\begin{array}{cc} \; & \beta_{j}\in B=\left\{\beta_{j}|\beta_{j}\in \left\{0,1\right\},\forall j\in C\right\} \end{array}$$

The objective function in P3 quantifies the incremental sum rate for all FD-DUs. This optimization ensures the best possible performance across the FD-DUs.

In order to address P3, the sets of vertices correspond to CUs and DUs, and the edge weights between these vertices are computed using $\Delta R_{i,j}^{C,u}$ and $\Delta R_{i,j}^{C,d}$. Consequently, this problem is transformed into a classical maximum-weight bipartite matching problem, which can be efficiently solved using the Kuhn–Munkres algorithm. The algorithm flow is presented in Algorithm 1. It’s important to note that in an actual network, $\Delta R_{i,j}^{C}$ should be less than or equal to zero due to the introduced interference after pairing. If $\Delta R_{i,j}^{C}$ is greater than zero, we consider it a D2D access failure.
**Algorithm 1** The optimal resource allocation algorithm for the sum FD-DUs maximization design  1:Initialize the rate variation matrix for FD-DUs ${\left\{\Delta R_{i,j}^{D}\right\}}_{S\times T}$ and the pairing indicator matrix ${\left\{\rho_{i,j}\right\}}_{S\times T}$.  2:**for** j=1:T **do**  3:  **for** i=1:S **do**  4:   Obtain the optimal power solution $\left(p_{i}^{opt},p_{j}^{opt}\right)$ from the power control algorithm in Section 3. B for the single pair CU*j*-DU*i*.  5:   Substitute $\left(p_{i}^{opt},p_{j}^{opt}\right)$ into (2)–(9), (20) and (21) to calculate the $\Delta R_{i,j}^{D}$ (including uplink and downlink).  6:   Set the $\rho_{i,j}=1$.  7:   **if** $\Delta R_{i,j}^{C}>0$ **then**  8:    DU*i* and CU*j* are pairing failure, FD-D2D is prohibited from accessing, CU maintains the original link, i.e., set    
$\Delta R_{i,j}^{C}=0$    
$R_{i,j}^{C}=R_{j}^{unp}$    
$R_{i,j}^{D}=0$    
$\rho_{i,j}=0$  9:   **end if**10:  **end for**11:**end for**12:Employ Kuhn–Munkres maximum-weight algorithm to get the optimal pattern ${\left\{\rho_{i,j}\right\}}_{S\times T}$ of ${\left\{\Delta R_{i,j}^{D}\right\}}_{S\times T}$.13:Return the optimal resource sharing pattern ${\left\{\rho_{i,j}\right\}}_{S\times T}$ and sum of $\Delta R_{i,j}^{D}$, which are picked by the pattern.

At this stage, the MaxSumDU-OP algorithm for maximizing the capacity of FD-DUs has been completed. In terms of computation complexity, the optimal power solution for a single pair is found within a finite set with a complexity of O(1). This results in a total complexity of O(ST) for the power control algorithm for all CU-DU pairs. Furthermore, for resource allocation,  Algorithm 1 solves the user pairing in $O(S^{3})$ time due to our scenario assumption that T≥S. Therefore, the overall complexity of MaxCU-OPOP is $O(ST+T^{3})$.

## 4. Individual FD-DU Capacity Uniform Design

The design goal of maximizing the sum capacity for FD-DUs can significantly improve overall D2D performance from the network operator’s perspective. However, it may lead to fairness concerns for individual DUs, especially those with weaker channel conditions. In such a design, DUs with favorable channel conditions tend to receive more resources, potentially at the expense of DUs with poorer channel conditions, which can result in a terrible user experience for some DUs. In order to address this fairness issue, we have introduced the MaxMinDU-OP algorithm. This algorithm aims to maximize the minimum rate among all FD-DUs in the cell, thereby promoting a more uniform and equitable user experience.

### 4.1. Problem Formulation

In order to maximize the minimum rate of all FD-DUs, the optimization problem can be formulated as P4.

(24) $$\begin{array}{cc} P4:\;\;\;\; & \max_{\begin{array}{c} \rho_{i,j},\;p_{i}\\ p_{j},\;p_{B,j} \end{array}}\;\min_{i\in D}2R_{i}\\ s.t.\;\;\;\;\; & \left(11a)-(11i\right) \end{array}$$

The max-min optimization problem in P4 is a special case of the weighted Chebyshev objective function, where all weights are set to one [22]. This allows us to transform it from a multi-objective optimization problem into a single-objective optimization problem while ensuring Pareto optimality [23]. In order to address P4, we decompose it into power control and resource allocation subproblems. The power control aspect is identical to that of the MaxCU-OPOP algorithm, where the optimal power solution maximizes $R_{i,j}^{D}$ for a single pair. Therefore, our focus in the following will be solely on resource allocation.

### 4.2. Resource Allocation

By incorporating the optimal power solution from P2, the original optimization problem in P4 can be represented as follows:

(25) $$\begin{array}{cc} P5:\;\;\;\; & \max_{\rho_{i,j}}\;\min_{i\in D}\sum_{j\in C}\rho_{i,j}R_{i,j}^{D} \end{array}$$

(25a) $$\begin{array}{cc} s.t.\;\;\;\;\; & \sum_{j\in C}\rho_{i,j}\leq 1,\;\rho_{i,j}\in \left\{0,1\right\},\;\forall i\in D \end{array}$$

(25b) $$\begin{array}{cc} \; & \sum_{i\in D}\rho_{i,j}\leq 1,\;\rho_{i,j}\in \left\{0,1\right\},\;\forall j\in C \end{array}$$

In order to efficiently address P5 with low computational complexity, we have developed a unified algorithm with polynomial time complexity. This algorithm comprises two essential components, making use of the Kuhn–Munkres method. In the initial part, we assess whether a selected threshold value, denoted as τ, exceeds the desired optimal minimum rate for all FD-DUs, as illustrated in Algorithm 2.
**Algorithm 2** Part 1 of the optimal resource allocation algorithm for P5  1:Give an arbitrary number τ function as a threshold value.  2:Initialize the rate matrix for paired CU ${\left\{R_{i,j}^{D}\right\}}_{S\times T}$ and an all-zero matrix $F_{S\times T}={\left\{F_{i,j}\right\}}_{S\times T}$.  3:**for** j=1:T **do**  4:  **for** i=1:S **do**  5:   Calculate the optimal power solution $\left(p_{i}^{opt},p_{j}^{opt}\right)$ through the power control algorithm in Section 3. B for the single pair CU*j*-DU*i*.  6:   Substitute $\left(p_{i}^{opt},p_{j}^{opt}\right)$ into (2)–(9) to get the $R_{i,j}^{D}$ (including uplink and downlink). The rest is the same as Algorithm 1.  7:   **if** $R_{i,j}^{D}<\tau$ **then**  8:    $F_{i,j}=1$  9:   **else**10:    $F_{i,j}=0$11:   **end if**12:  **end for**13:**end for**14:Utilize Kuhn–Munkres minimum-weight algorithm for F and return the minimum total cost, denoted as *k*.15:**if** k=0 **then**16:  It represents that no elements are smaller than τ in this assignment, meaning that τ is less than or equal to the desired minimum rate across all FD-DUs.17:**else if** k>0 **then**18:  It indicates that there is no assignment that ensures all elements are greater than or equal to τ, signifying that τ exceeds the desired minimum capacity.19:**end if**

Steps 16 and 18 in Algorithm 2 are crucial conclusions for Algorithm 2. Building on these steps, we have designed a second uniform resource allocation algorithm. This algorithm begins by arranging all elements of the DU rate matrix ${\left\{R_{i,j}^{D}\right\}}_{S\times T}$ into a vector, totaling S·T elements. It then proceeds to locate the position of the minimum rate through a bisection search. Once the bisection search is completed, the Kuhn–Munkres minimum-weight algorithm provides an assignment that maximizes the minimum rate across all FD-DUs. The detailed process of the second part is illustrated in Algorithm 3.

The computational load of the proposed uniform algorithm primarily stems from three key aspects: the generation of $\{R_{i,j}^{D}\}S\times T$, the sorting of elements in ${\left\{R_{i,j}^{D}\right\}}_{S\times T}$, and the bisection search for the optimal value using the Kuhn–Munkres method. Their complexities are O(ST), O(ST·logST), and $O(S^{3}\cdot \log ST)$, respectively, assuming that T≥S. Consequently, the overall computational complexity of the uniform algorithm for CUs is $O(ST+ST\cdot \log ST+S^{3}\cdot \log ST)$.
**Algorithm 3** Part 2 of the optimal resource allocation algorithm for P5  1:Obtain the rate matrix for the accessed DU ${\left\{R_{i,j}^{D}\right\}}_{S\times T}$ from Algorithm 2.  2:Initialize the element index m=1 and n=S·T.  3:Sort all elements of ${\left\{R_{i,j}^{D}\right\}}_{S\times T}$ into a vector v in ascending order.  4:**if** S=1 **then**  5:  $R_{i,j}^{D,min}=v\left(T\right)$  6:  Locate v(T) in ${\left\{R_{i,j}^{D}\right\}}_{1\times T}$ and set the corresponding $\rho_{i,j}=1$, otherwise $\rho_{i,j}=0$.  7:  Return the maximization of the minimum cellular rate $R_{i,j}^{D,min}$ and the optimal uniform resource allocation pattern ${\left\{\rho_{i,j}\right\}}_{1\times T}$.  8:**end if**  9:**while** (n−m)>1 **do**10:  l=(n−m)/2;11:  ${\left\{F_{i,j}\right\}}_{S\times T}=0_{S\times T}$;12:  **for** j=1:T **do**13:   **for** i=1:S **do**14:    **if** $R_{i,j}^{D}<v\left(l\right)$ **then**15:     $F_{i,j}=1$;16:    **else**17:     $F_{i,j}=0$;18:    **end if**19:   **end for**20:  **end for**21:  Employ the Kuhn–Munkres minimum-weight algorithm for ${\left\{F_{i,j}\right\}}_{S\times T}$ to yield the assignment denoted as $E_{Q\times P}$ and the corresponding lowest total cost denoted as *k*.22:  **if** k>0 **then**23:   n=l;24:  **else**25:   m=l;26:   ${\left\{\rho_{i,j}\right\}}_{S\times T}=E$;27:  **end if**28:**end while**29:Locate the minimum rate in ${\left\{R_{i,j}^{D}\cdot \rho_{i,j}\right\}}_{S\times T}$, excluding the zero element, denoted as $R_{i,j}^{D,min}$.30:Return the maximization of minimum cellular rate $R_{i,j}^{D,min}$ and the optimal uniform resource allocation pattern ${\left\{\rho_{i,j}\right\}}_{S\times T}$.

## 5. Numerical Results Analysis

In this section, we present the numerical results to validate our two proposed algorithms for an FD-D2D underlying cellular network. The system configuration is illustrated in Section 2. Specifically, there are T CUs and S FD-D2D pairs randomly distributed within the cell, with the BS located at the center. The FD-D2D pairs have the flexibility to choose both uplink and downlink CUs for spectrum sharing based on the interference management algorithm. In order to ensure robustness, the two algorithms proposed in this paper have been simulated by Monte Carlo methods, with simulations conducted over 10,000 iterations to account for randomness. The key parameters used in our simulation are outlined in Table 2. We adopted a self-interference cancellation capability of 110 dB, as it represents a conservative option and is relatively easy to achieve. However, it is worth mentioning that recent papers have reported capabilities as high as 125 dB [11,12,13].

We assume an equal spectrum assignment for each CU, with one subcarrier allocated to each CU. The transmit power from the BS is uniformly distributed across the entire bandwidth, resulting in the transmit power from the BS to CU*j* as $P_{B,j}=P_{\mathrm{BS}}^{\max}/K$.

**Figure 3:**
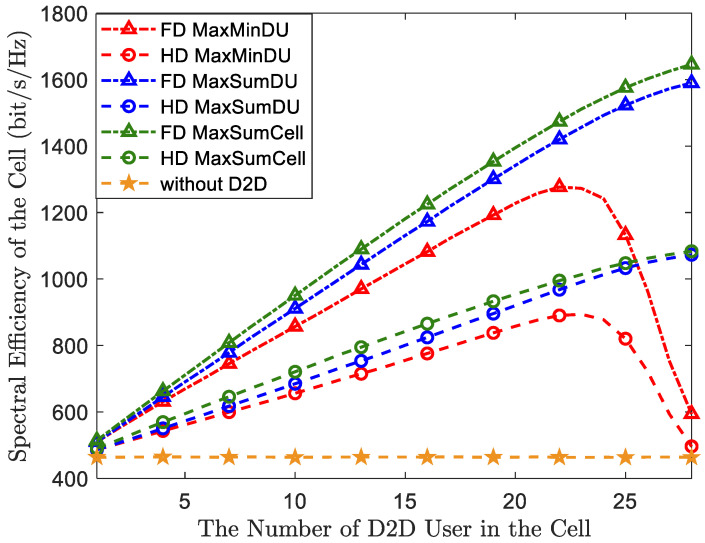
System SE comparison between the FD and HD D2D underlying networks with respect to the growing number of DUs.

In order to assess the advantages of incorporating full-duplex (FD) characteristics into the D2D system from a system perspective, we compare an FD-D2D underlying network with the traditional half-duplex (HD) D2D system using the two algorithms. We evaluate the cell’s sum rate in Figure 3, which illustrates the sum rate of the entire cell achieved by our model with the two proposed algorithms as the number of DUs increases. The traditional HD-D2D underlying network serves as an ideal benchmark. It is observed that both the FD and HD modes of D2D can improve the system spectral efficiency (SE) compared to a cellular network without D2D. Specifically, when employing the MaxSumDU-OP algorithm and the MaxMinDU-OP algorithm in the FD mode, the system SE improves by approximately 48% and 20%, respectively, compared to the HD mode when the number of DUs is equal to the number of CUs.

When comparing the proposed algorithms in Figure 3, the network employing the MaxSumDU-OP algorithm closely resembles the network employing the MaxMinDU-OP algorithm when the number of DUs is less than or equal to 22, regardless of whether D2D is in FD mode or HD mode. However, as the number of DUs continues to grow, the system SE of the MaxMinDU-OP algorithm starts to significantly degrade when there are more than 22 DUs. In contrast, the growth rate of the MaxSumDU-OP algorithm remains almost constant. This difference can be attributed to the fact that as the number of DUs in the cell increases, more mutual interference is introduced. The system performance of the MaxMinDU-OP algorithm becomes more sensitive than that of the MaxSumDU-OP algorithm to the increasing mutual interference when there are more than 22 DUs. This is because the BS has to allocate more resources and power to the DU with the minimum rate, which is in the worst interference environment, in order to act against interference to maximize its performance. Furthermore, we introduce the MaxSumCell-OP algorithm in Figure 3, the objective of which is maximizing the sum rate of the cell by employing the power control and resource allocation method in Section 3, functioning as a benchmark to compare it against the proposed algorithm. The comparison between MaxSumCell-OP and MaxSumDU-OP indicates that the MaxSumDU-OP algorithm sacrifices a little system performance to ensure FD-D2D link throughput maximization.

**Figure 4:**
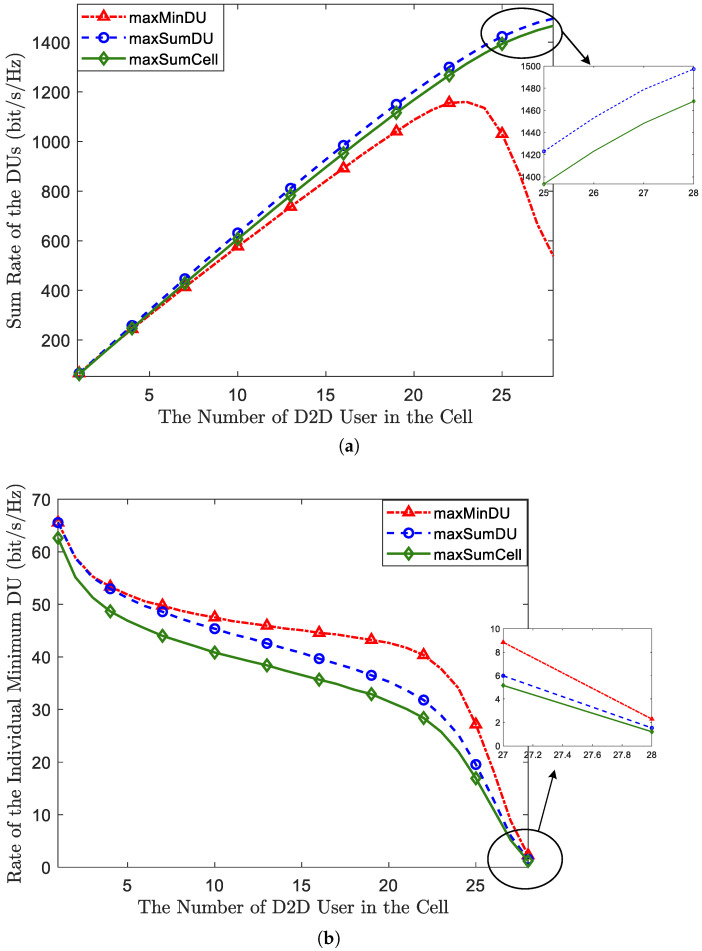
The validation of effectiveness for the proposed algorithm. (**a**) The comparison of the sum rates of the DUs; (**b**) the comparison of the minimum rates of the DUs.

Then, we validate the effectiveness of the two proposed algorithms by utilizing two metrics: the sum rate of DUs and the minimum rate of DUs, as per Figure 4, and the FD-D2D underlying network employing the MaxSumCell-OP algorithm serves as the benchmark. Figure 4a demonstrates the comparison of the sum rates of the DUs with respect to the increasing number of DUs. We can observe that, in terms of the sum rate of the DUs, MaxSumDU-OP is a little superior to MaxSumCell-OP, and they both surpass the MaxMinDU-OP algorithm, especially when the number of DUs is greater than 22. This result indicates that the proposed MaxSumDU-OP can further improve D2D link throughput effectively, although this improvement is limited. The sudden drop at DU >22 will be explained in a numerical analysis of the access rate. As for a comparison of the minimum rate of the DUs, Figure 4b shows that MaxMinDU-OP is strictly above MaxSumCell-OP and MaxSumDU-OP in terms of the minimum rate of the DUs, which means the algorithm has worked effectively. However, it is worth pointing out that the minimum DU rate of these three algorithms gradually declines as the number of DUs increases; when the DUs reach the maximal value, MaxMinDU-OP only slightly outperforms the other two algorithms. Additionally, when DU ⩽4, MaxSumDu-OP is very close to MaxMinDU-OP. The reason is that maximizing the sum rate and maximizing the minimum rate for DUs is almost the same objective in the scenarios where there are a few DUs.

**Figure 5:**
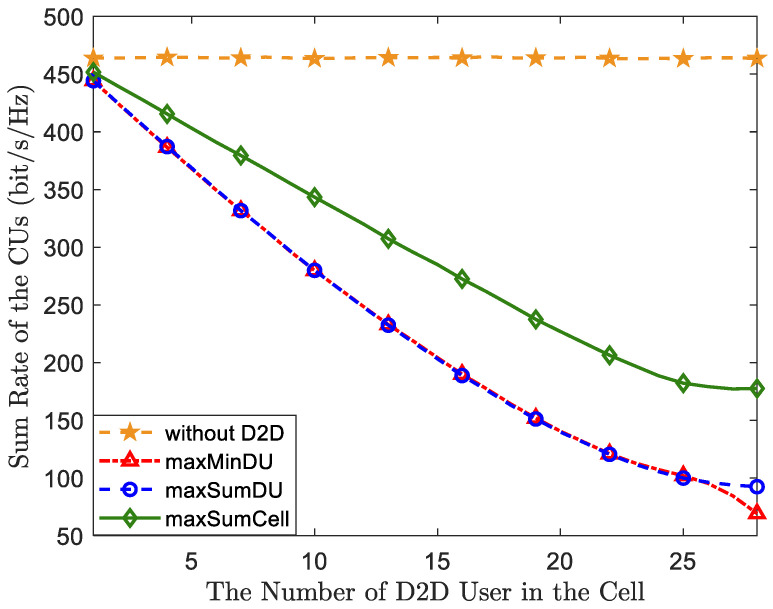
A comparison of the two proposed algorithms compared to MaxCell-OPOP.

After analyzing the effectiveness, we realized that the BS utilizes the two proposed algorithms to ensure the performance of DUs by allocating more resources and power to them than to CUs. This also implies sacrificing the performance of the CUs, as well as compromising the overall performance of the cell, which has been analyzed in Figure 3. We illustrate the trade-off of the proposed algorithms compared to a cellular network without D2D and the MaxSumCell-OP algorithm by using the sum rate of CUs in Figure 5. It can be observed that in Figure 5, as long as D2D is introduced, the rate of CU decreases by a significant margin. The curves of the two proposed algorithms almost overlap, and the sum CU rate compromised by MaxSumCell-OP is less than that sacrificed by the two proposed algorithms. This observation indicates that more resources need to be allocated to DUs to ensure their optimal performance compared to a system rate maximization design. From another perspective, in certain scenarios, the system may aim to offload more traffic from the CUs to DUs. In this context, we can also consider that MaxSumDu-OP and MaxMinDU-OP can more effectively alleviate the pressure on the cellular system while improving overall cell throughput.

**Figure 6:**
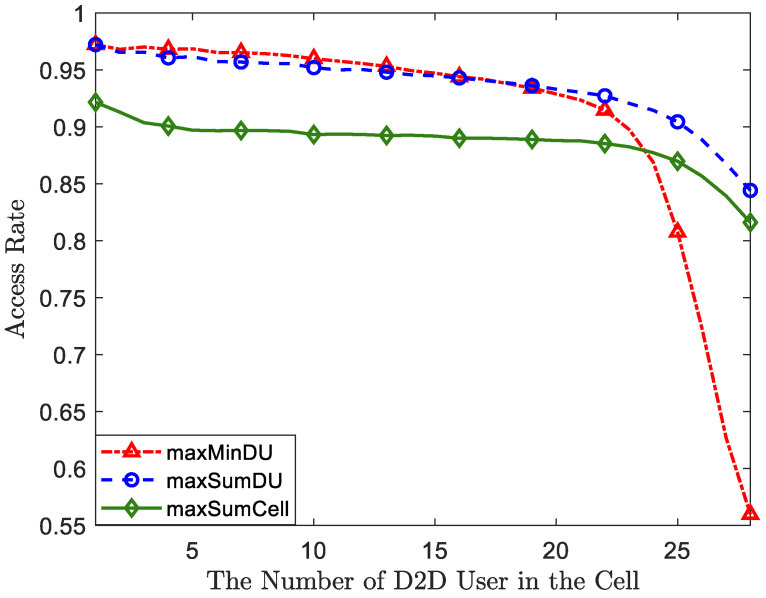
A comparison of the access rates with respect to the growing number of DUs.

In order to ensure user experience, we have considered quality of service (QoS) as a threshold. When spectrum sharing between CUs and DUs results in rates that fall below the threshold, the BS restricts FD-D2D access. Therefore, the access rate of D2D is a crucial metric for evaluating our algorithms. Figure 6 shows the access rate of the DUs with a growing number of DUs for the proposed algorithms compared with MaxSumCell-OP. From the figure, these three algorithms all experience a decrease in access rate as the number of DUs increases. MaxSumDU-OP is strictly greater than MaxSumCell-OP, which indicates that the performance protection of the DUs has worked. The curve of MaxMinDU-OP nearly overlaps with MaxSumDU-OP when the number of DUs is less than or equal to 22. In order to ensure a satisfactory user experience, we have taken quality of service (QoS) into account as a threshold. When spectrum sharing between CUs and DUs results in rates falling below this threshold, the BS restricts FD-D2D access. Therefore, the access rate of D2D communication is a crucial metric for evaluating our algorithms. Figure 6 illustrates the access rate of DUs as the number of DUs increases for the proposed algorithms compared to MaxSumCell-OP. From the figure, we can observe that all three algorithms experience a decrease in access rate as the number of DUs increases. MaxSumDU-OP consistently outperforms MaxSumCell-OP, indicating the effectiveness of our performance protection for the DUs. The curve for MaxMinDU-OP closely aligns with that of MaxSumDU-OP when the number of DUs is less than or equal to 22. However, when the number of DUs continues to grow, the access rate of MaxMinDU-OP has a dramatic drop and declines below MaxSumCell-OP when the number of DUs equals 24. When the DUs approach the full load, the access rate of MaxMinDU-OP drops to only 56%. This phenomenon precisely explains the significant drop in the performance of the MaxMinDU-OP algorithm in Figure 3 and Figure 4a. Since increasing interference makes the performance of D2D fail to fulfill the QoS, the prohibited access directly results in performance degradation. Therefore, we do not recommend using the MaxMinDU-OP algorithm when the number of DUs surpasses three-quarters of the full load.

## 6. Conclusions

In this paper, we have introduced two algorithms aimed at ensuring the performance of FD-D2D links in cellular networks. The first algorithm, MaxSumDU-OP, focuses on maximizing the sum rate of FD-DUs, thereby guaranteeing the overall performance of the entire group of DUs. The second algorithm, MaxMinDU-OP, is designed to maximize the minimum rate across FD-DU pairs, with the goal of providing uniform performance for individual FD-DU pairs. Formulating these optimization problems as a MINLP presents mathematical intractability. In order to address this challenge, we have decomposed the optimization problem into two parts: power control, which is solved using nonlinear programming, and resource allocation, for which we utilize the Kuhn–Munkres method.

We have compared the performance of the proposed algorithms against a cellular network without D2D communication and the MaxSumCell-OP algorithm, which aims to maximize the overall rate for the entire cell and serves as a benchmark. The numerical results validate the effectiveness of the proposed algorithms. Specifically, MaxSumDU-OP performs well across the whole range of the number of DUs, while MaxMinDU-OP is effective when the number of DUs is less than three-quarters of the full load. These algorithms strike a balance between the sum rate of the cell and the sum rate of CUs to ensure the performance of FD-DUs. Therefore, the choice of an algorithm should be based on the specific scenario and requirements. It is worth noting that our current work is limited to “one-to-one” spectrum sharing scenarios, laying a solid foundation for studying more complex sharing methods in future research.

## Figures and Tables

**Table 1:** The value of major parameters in our simulation.

Notation	Definition
$\beta_{j,B}$	the gain of fast fading with an exponential distribution
$\Gamma_{j,B}$	the gain of slow fading with a log-normal distribution
*G*	the path loss constant
α	the path loss exponent
$l_{j,B}$	the distance between CU*j* and the BS

**Table 2:** The value of major parameters in our simulation.

Parameter	Value
Cell radius	500 m
Fast fading	mean = 1
Slow fading	standard deviation = 8 dB
Pathloss exponent (α)	3
Pathloss constant (*G*)	$10^{-2}$
Noise spectral density ($N_{0}$)	−174 dBm/Hz
Users distribution	Uniform
$P_{j}^{\max\;}$,$P_{i}^{\max\;}$	24 dBm
$P_{BS}^{\max\;}$	46 dBm
Number of CUs (*T*)	28 (50% uplink & 50% downlink)
Number of DUs (*S*)	0 to 28
Self-interference cancellation	110 dB
Bandwidth	10 MHz
Number of subcarriers (*K*)	20
D2D distance (*d*)	8 m
$\gamma_{j}^{d,\mathrm{req}}$,$\gamma_{j}^{u,\mathrm{req}}$,$\gamma_{i}^{\mathrm{req}}$	10 dB

## Data Availability

Data are contained within the article.
